# Effects of CDP-Choline and Choline on Gastrointestinal Inflammatory and Monoaminergic Responses in an LPS-Induced Rat Endotoxemia Model

**DOI:** 10.3390/biomedicines14102271

**Published:** 2026-10-08

**Authors:** Shideh Roshani, Elif Baris, Emre Hamurtekin, Candan Ozogul, Ahmet Sami Bosnak

**Affiliations:** 1 Faculty of Pharmacy, Cyprus International University, 99258 Nicosia, Türkiye; 2 Department of Medical Pharmacology, Faculty of Medicine, Izmir University of Economics, 35330 İzmir, Türkiye; 3 Department of Pharmacology, Faculty of Pharmacy, Eastern Mediterranean University, 99628 Famagusta, Türkiye; 4 Department of Histology, Faculty of Medicine, University of Kyrenia, 99300 Kyrenia, Türkiye; 5 Department of Clinical Pharmacy, Faculty of Pharmacy, Cyprus International University, 99258 Nicosia, Türkiye

**Keywords:** endotoxemia, CDP-choline, choline, gastrointestinal inflammation, monoamines

## Abstract

**Objectives**: Endotoxemia is associated with pronounced gastrointestinal (GI) inflammatory responses and disruption of local neurochemical regulation. Although cytidine-5′-diphosphocholine (CDP-choline) and choline exert anti-inflammatory effects, their region-specific actions on GI inflammation and monoaminergic alterations during endotoxemia remain poorly characterized. **Methods**: Acute endotoxemia was induced in female Sprague–Dawley rats by intraperitoneal lipopolysaccharide (LPS; 10 mg/kg). Endotoxemia severity was assessed using a standardized sepsis scoring system at 6 and 24 h. GI tissues (esophagus, stomach, duodenum, cecum, and colon) were analyzed for tumor necrosis factor-α (TNF-α), interleukin-6 (IL-6), choline acetyltransferase (ChAT), serotonin (5-HT), dopamine (DA), and norepinephrine (NE) by ELISA, alongside histopathological evaluation. **Results**: LPS significantly elevated sepsis scores, cytokine levels, and monoamine concentrations across all GI regions, with the most pronounced cytokine responses in the duodenum, cecum, and esophagus. CDP-choline attenuated TNF-α and IL-6 elevations more effectively than choline; markedly increased ChAT levels, especially in the esophagus, cecum, and colon; and reduced LPS-induced 5-HT and DA elevations in a region-dependent manner. NE responses were heterogeneous across regions. Histopathologically, CDP-choline largely preserved normal tissue architecture throughout the GI tract, whereas choline provided only partial protection in intestinal tissues. **Conclusions**: CDP-choline exerted stronger modulatory effects than choline on region-specific GI inflammatory, cholinergic, and monoaminergic responses during acute endotoxemia, supporting further investigation of cholinergic precursors as modulators of GI neuroimmune dysfunction in systemic inflammation.

## 1. Introduction

Sepsis is defined as life-threatening organ dysfunction caused by a dysregulated host response to infection. Its pathophysiology involves concurrent inflammatory and immunosuppressive responses rather than excessive inflammation alone. Endotoxemia refers to the presence of bacterial endotoxin, principally lipopolysaccharide (LPS), in the circulation and is distinct from sepsis. LPS, a major component of the outer membrane of gram-negative bacteria, activates innate immune responses and promotes the release of inflammatory mediators. Experimental administration of purified LPS induces an acute systemic inflammatory response without introducing viable pathogens or establishing an ongoing infection. This approach allows selected inflammatory and tissue responses to be investigated under controlled conditions, but it does not reproduce the evolving host–pathogen interactions or clinical heterogeneity of infection-associated sepsis. Accordingly, LPS challenge is more accurately described as a model of experimental endotoxemia and acute systemic inflammation than as a model of sepsis itself. In the present study, this model was used to investigate the effects of CDP-choline and choline on gastrointestinal inflammatory and neuroendocrine responses to an endotoxin challenge. LPS is recognized primarily through the CD14–MD-2–TLR4 receptor complex, triggering downstream inflammatory signaling pathways, including NF-κB activation and production of pro-inflammatory mediators such as TNF-α, IL-1β, and IL-6 [1].

The gastrointestinal (GI) tract is an important target of endotoxemia and may also contribute to systemic inflammatory responses through increased cytokine production and disruption of local immune homeostasis [2,3]. Due to its extensive immune cell population and dense enteric nervous system, the GI tract plays a central role in inflammatory responses. The relationship between gastrointestinal inflammation and local neurochemical alterations during endotoxemia can be investigated by examining inflammatory and neurochemical markers across different GI regions. In this study, tissue TNF-α and IL-6 levels were assessed alongside choline acetyltransferase (ChAT), an enzyme involved in acetylcholine synthesis, and the monoamines serotonin (5-HT), dopamine (DA), and norepinephrine (NE). Comparing these measurements across the esophagus, stomach, duodenum, cecum, and colon allows characterization of regional inflammatory and neurochemical responses to LPS and their modulation by CDP-choline and choline, without directly establishing functional changes in neurotransmission [4,5].

Cholinergic signaling represents an important endogenous regulatory mechanism controlling inflammatory responses during endotoxemia. The cholinergic anti-inflammatory pathway (CAP) suppresses cytokine production primarily through activation of alpha-7 nicotinic acetylcholine receptors expressed on immune cells. Stimulation of these receptors inhibits nuclear factor kappa B signaling and reduces the release of pro-inflammatory cytokines, highlighting the relevance of cholinergic mechanisms in inflammation control [6]. Cholinergic regulation of inflammatory responses provides a rationale for investigating choline and CDP-choline in experimental endotoxemia [7]. However, their effects on inflammatory and neurochemical profiles across different gastrointestinal regions remain insufficiently characterized. Assessing tissue inflammatory cytokines alongside ChAT and monoamines may help clarify whether these compounds exert region-dependent effects during acute systemic inflammation. In GI tissues, cholinergic activity depends on local acetylcholine (ACh) synthesis, which is mediated by ChAT enzyme. Changes in ChAT may therefore be associated with modulation of local inflammatory responses, as well as a stable indicator of local cholinergic capacity [8].

Cytidine-5′-diphosphate-choline (CDP-choline) is an endogenous intermediate in phosphatidylcholine biosynthesis and is rapidly metabolized to choline and cytidine following administration. CDP-choline increases choline availability and supports membrane phospholipid synthesis through the Kennedy pathway. Experimental studies have demonstrated that CDP-choline attenuates inflammatory responses during endotoxemia, reduces cytokine production, and improves tissue integrity in several organ systems [5,7]. As a choline donor, CDP-choline and choline itself serve as a direct precursor for ACh synthesis, potentially contributing to CAP activation via cholinergic receptor interactions [7].

In addition to cytokine regulation, LPS-induced inflammation affects enteric monoamines, including 5-HT, DA, and NE, which play key roles in GI motility, secretion, and immune regulation. Inflammatory cytokines can influence 5-HT transporter activity and alter tissue serotonin availability [9]. Experimental studies have shown that cholinergic agonists modify 5-HT levels during endotoxic conditions [10], while LPS exposure may reduce 5-HT availability through transporter-mediated mechanisms [11].

Dopaminergic signaling within GI tissues is also sensitive to inflammatory conditions. LPS has been reported to alter tissue DA levels in experimental models, with both reductions and compensatory increases described depending on the tissue and timing of exposure [12,13]. Choline supplementation has been associated with modulation of dopaminergic availability in inflammatory and metabolic conditions [14], suggesting interactions between cholinergic and monoaminergic systems during endotoxemia challenge.

Previous studies have demonstrated anti-inflammatory and tissue-protective effects of CDP-choline in experimental sepsis. Sevim et al. reported reduced circulating inflammatory cytokines and protection against injury in several organs [15], while Tihan et al. demonstrated attenuation of liver and small-intestinal morphological damage in a cecal ligation and puncture model [16]. Barış et al. also investigated CDP-choline and choline in LPS-induced inflammation, focusing on cyclooxygenase expression, prostaglandins, and circulating TNF-α [6]. The present study extends these investigations by comparing CDP-choline and choline across five gastrointestinal regions—the esophagus, stomach, duodenum, cecum, and proximal colon—through the combined assessment of tissue TNF-α and IL-6, ChAT as a marker of cholinergic synthetic capacity, serotonin, dopamine, norepinephrine, and histopathological changes.

## 2. Materials and Methods

This experimental study used 6–8-week-old female Sprague–Dawley rats weighing 180–240 g. This experimental study used 6–8-week-old female Sprague–Dawley rats weighing 180–240 g, obtained from the Eastern Mediterranean University Faculty of Pharmacy Experimental Animal Research and Application Center (DAÜ-EFDAM), Famagusta, North Cyprus. All animal procedures were approved by the University of Kyrenia Local Ethics Committee for Animal Experiments, Kyrenia, North Cyprus (decision no. 01, dated 17 December 2025; approval letter no. ETK25/GD-004, dated 18 December 2025). Animal care followed the institutional standards of the Eastern Mediterranean University Faculty of Pharmacy Experimental Animal Research and Application Center (DAÜ-EFDAM). Animals were allowed to acclimatize to the facility for 7 days before experimental procedures. Rats were housed individually in conventional open-top polypropylene cages with wire-bar lids at 22 ± 2 °C and 45–65% relative humidity under a 12 h light/dark cycle, with free access to standard pellet chow and water. Environmental enrichment was provided in the form of nesting material; no other enrichment items were provided. Bedding was changed at least twice weekly. General health, behavior, and feeding were monitored daily. Clinical severity was assessed at baseline and at 6 and 24 h after LPS administration using a standardized scoring system, and body weight was recorded at baseline and 24 h. At 24 h, cardiac blood collection was performed under deep anesthesia induced by intraperitoneal ketamine (80 mg/kg) and xylazine (10 mg/kg), followed by cervical dislocation while the animals remained deeply anesthetized.

Female rats were used to evaluate endotoxemia-related inflammatory responses in a sex that remains relatively underrepresented in experimental studies, despite evidence that immune, neuroendocrine, and cholinergic signaling may differ between sexes [17,18].

### 2.1. Experimental Protocol

Systemic inflammation was induced by a single intraperitoneal injection of a sublethal dose of LPS to induce acute endotoxemia for 24 h (10 mg/kg). Animals were randomly allocated to four experimental groups (*n* = 6 per group): a saline-treated control group (0.9% NaCl (saline)); LPS group; LPS + CDP-choline (375 mg/kg, i.p.); and LPS + choline groups (90 mg/kg, i.p.). CDP-choline or choline was administered 5 min before and 6 h after LPS injection, while control and LPS groups received 0.9% NaCl (saline) at the same time points as previously described. Lipopolysaccharide from *Escherichia coli* O55:B5 was obtained from Med Chem Express (Monmouth Junction, NJ, USA; catalog no. HY-D1056). The powder was stored at +4 °C until use. A fresh stock solution was prepared on each experimental day by dissolving 10 mg of LPS in 1 mL of 0.9% NaCl. For each rat, the volume required to deliver 10 mg/kg was calculated according to body weight and diluted with 0.9% NaCl to a final injection volume of 1 mL. LPS was administered as a single intraperitoneal injection. CDP-choline or choline was administered in separate intraperitoneal injections 5 min before and 6 h after LPS administration; neither treatment was mixed with LPS in the same syringe. CDP-choline sodium salt dihydrate and choline chloride obtained from Sigma-Aldrich (St. Louis, MO, USA) were administered intraperitoneally at 375 and 90 mg/kg, respectively, doses selected to deliver approximately equimolar amounts of choline moiety (~71.5 vs. ~67.1 mg/kg choline base), based on the dosing regimen of Barış et al. [6]. Based on molecular weights of 546.34 g/mol for CDP-choline sodium salt dihydrate and 139.62 g/mol for choline chloride, these doses correspond to theoretical choline-moiety amounts of approximately 71.5 and 67.1 mg/kg per injection, respectively. Thus, the regimens provided approximately comparable choline content but were not strictly equimolar. Comparable choline content does not establish equivalent systemic or gastrointestinal tissue exposure. This regimen, therefore, represents a primarily preventive intervention with repeated dosing after endotoxin exposure, rather than treatment initiated after the onset of endotoxemia.

Endotoxemia severity was assessed using standardized sepsis scoring system blindly at baseline, 6 h, and 24 h, as previously described [19]. Animals were identified by codes, and the assessor was blinded to treatment allocation during all scoring sessions. Body weight was recorded at baseline and 24 h after LPS administration to assess systemic responses to endotoxemic challenge. The experimental protocol ended 24 h after LPS administration, and rats were sacrificed with ketamine (80 mg/kg) and xylazine (10 mg/kg). GI tissues (esophagus, stomach, duodenum, cecum, and proximal colon) were rapidly excised, flash-frozen in liquid nitrogen, and stored in liquid nitrogen until subsequent biochemical, monoaminergic, and histopathological analyses. Clinical severity scoring and microscopic histopathological assessment were performed by assessors who were unaware of treatment allocation. No formal coding system was used for either assessment.

### 2.2. Histopathological Analyses

Samples from different gastrointestinal regions were fixed in 10% neutral buffered formalin, routinely processed, embedded in paraffin, sectioned at 5 µm, and stained with hematoxylin–eosin (H&E). Sections were examined under blinded conditions to assess inflammatory and structural alterations. Histopathological evaluation focused on inflammatory cell infiltration in the esophagus, epithelial vacuolization and erosive changes in the stomach, inflammatory cell infiltration in the duodenum and cecum, and inflammatory infiltration, hemorrhage, and epithelial erosion in the colon. Inflammatory cell subtypes were not specifically identified using H&E staining. Each parameter was scored semiquantitatively as 0 (absent), 1 (mild), 2 (moderate), or 3 (severe), and the mean score for each tissue was calculated [20]. Histopathological changes were classified as absent, mild, moderate, or severe. Mild inflammatory infiltration was defined as a slight increase in scattered inflammatory cells within the mucosa; moderate infiltration as more numerous inflammatory cells forming multifocal aggregates; and severe infiltration as dense, extensive inflammatory infiltrates associated with disruption of normal tissue architecture. Epithelial erosion was classified as mild when superficial epithelial loss was focal, moderate when multiple discrete areas of superficial epithelial loss were present, and severe when epithelial loss involved extensive, confluent areas. Examination was performed blinded to treatment allocation. Each slide contained one longitudinal tissue section evaluated at 40× magnification, with five microscopic fields assessed per section. One overall histopathological score was assigned per animal, which served as the experimental unit. Scores were compared among treatment groups within each tissue. Cellular vacuolization was classified as mild when occasional epithelial cells contained small cytoplasmic vacuoles, moderate when multiple groups of epithelial cells showed conspicuous vacuolization, and severe when widespread; prominent vacuolization was associated with marked cellular swelling. Absence of each lesion was recorded when no corresponding abnormality was detected [20,21,22].

### 2.3. ELISA Analyses

For ELISA analyses, 50 mg of GI tissue from each animal was individually homogenized in phosphate-buffered saline (PBS). For ELISA analyses, 50 mg of tissue from each gastrointestinal region of each animal was homogenized separately in 0.5 mL of PBS, maintaining a constant tissue-to-buffer ratio of 1:10 (*w*/*v*) across all samples. All samples were analyzed using commercial ELISA kits from Bioassay Technology Laboratory (Shanghai Korain Biotech Co., Ltd., Shanghai, China), including choline acetyltransferase (ChAT; catalog no. E0725Ra), interleukin-6 (IL-6; catalog no. E0135Ra), tumor necrosis factor-α (TNF-α; catalog no. E0764Ra), dopamine (DA; catalog no. EA0111Ra), serotonin (5-HT; catalog no. E0866Ra), and norepinephrine (NE; catalog no. E1137Ra), and were quantified according to the manufacturers’ instructions. Each tissue homogenate from each animal was analyzed in technical triplicate for each biomarker. These triplicates represented repeated measurements of the same biological sample. The three measurements were averaged to obtain one value per biomarker per tissue per animal before statistical analysis.

### 2.4. Statistical Analysis

Statistical analyses were performed using GraphPad Prism 10.2 (GraphPad Software, Boston, MA, USA). The individual animal was the experimental unit and biological replicate, with six independent animals per experimental group (*n* = 6; 24 animals in total). Each ELISA sample was analyzed in technical triplicate, and the three measurements were averaged to obtain one value per biomarker per gastrointestinal region per animal before statistical analysis. Technical measurements and different tissues collected from the same animal were not treated as additional independent biological replicates. Each biomarker was analyzed separately within each gastrointestinal region, and sepsis scores were analyzed separately at each time point, with treatment group as the sole comparison factor in each analysis. The distribution of data was assessed using the Shapiro–Wilk normality test. Comparisons among groups were conducted using one-way ANOVA with Tukey’s post hoc test for normally distributed data or the Kruskal–Wallis test followed by Dunn’s post hoc test for non-parametric data. A *p*-value < 0.05 was considered statistically significant, and results were expressed as mean ± SEM calculated from individual animal values.

## 3. Results

### 3.1. Effects of Choline and CDP-Choline on Endotoxemia Severity

Baseline body weights were comparable across groups prior to intervention. At 24 h, body weight remained stable in the saline group, showing no significant change from baseline (233.3 ± 14.0 g vs. 234.2 ± 13.4 g), corresponding to a slight weight gain (+0.9 ± 1.6 g). In contrast, the LPS group significantly reduced body weight, decreasing from 223.3 ± 16.7 g at baseline to 210.0 ± 15.3 g at 24 h (−13.3 ± 2.7 g, *p* < 0.01 vs. control). Baseline body weights did not differ significantly among groups (*p* = 0.32), although the LPS + CDP-choline group showed a lower mean and wider spread (186.6 ± 27.9 g) than the other groups, reflecting inter-animal variability at this sample size (*n* = 6) rather than a systematic difference between groups. The LPS + choline group showed only a slight, non-significant attenuation of body weight loss compared with the LPS group (203.3 ± 11.4 g to 200.0 ± 19.7 g; −3.3 ± 7.2 g, *p* > 0.05).

At both 6 h and 24 h, sepsis scores remained 0 in the control group. LPS administration caused a marked increase in sepsis scores at both time points (** *p* < 0.01 vs. control). At 6 h, neither CDP-choline nor choline significantly reduced LPS-induced sepsis scores. However, at 24 h, CDP-choline and choline significantly reduced the LPS-induced increase in sepsis scores (*p* < 0.01 and *p* < 0.05 vs. LPS, respectively). The effects of CDP-choline and choline on individual sepsis parameters, including appearance, level of consciousness, activity, response to stimulus, eye appearance, respiratory rate, and respiratory quality, together with total sepsis scores (Table 1).

### 3.2. Histopathological Analyses

Histopathological scoring demonstrated region-specific gastrointestinal inflammation following LPS administration. Compared with the control group, LPS significantly increased inflammation scores in the colon (* *p* < 0.05) and in the duodenum and cecum (** *p* < 0.01). In the stomach, the overall group comparison was significant (*p* = 0.020), but no pairwise comparison reached significance after correction, and no significant differences were found in the esophagus. CDP-choline significantly reduced inflammation scores compared with LPS in the duodenum, cecum, and colon (†† *p* < 0.01), whereas choline significantly reduced scores in the cecum and colon († *p* < 0.05) (Table 2).

**Figure 1:**
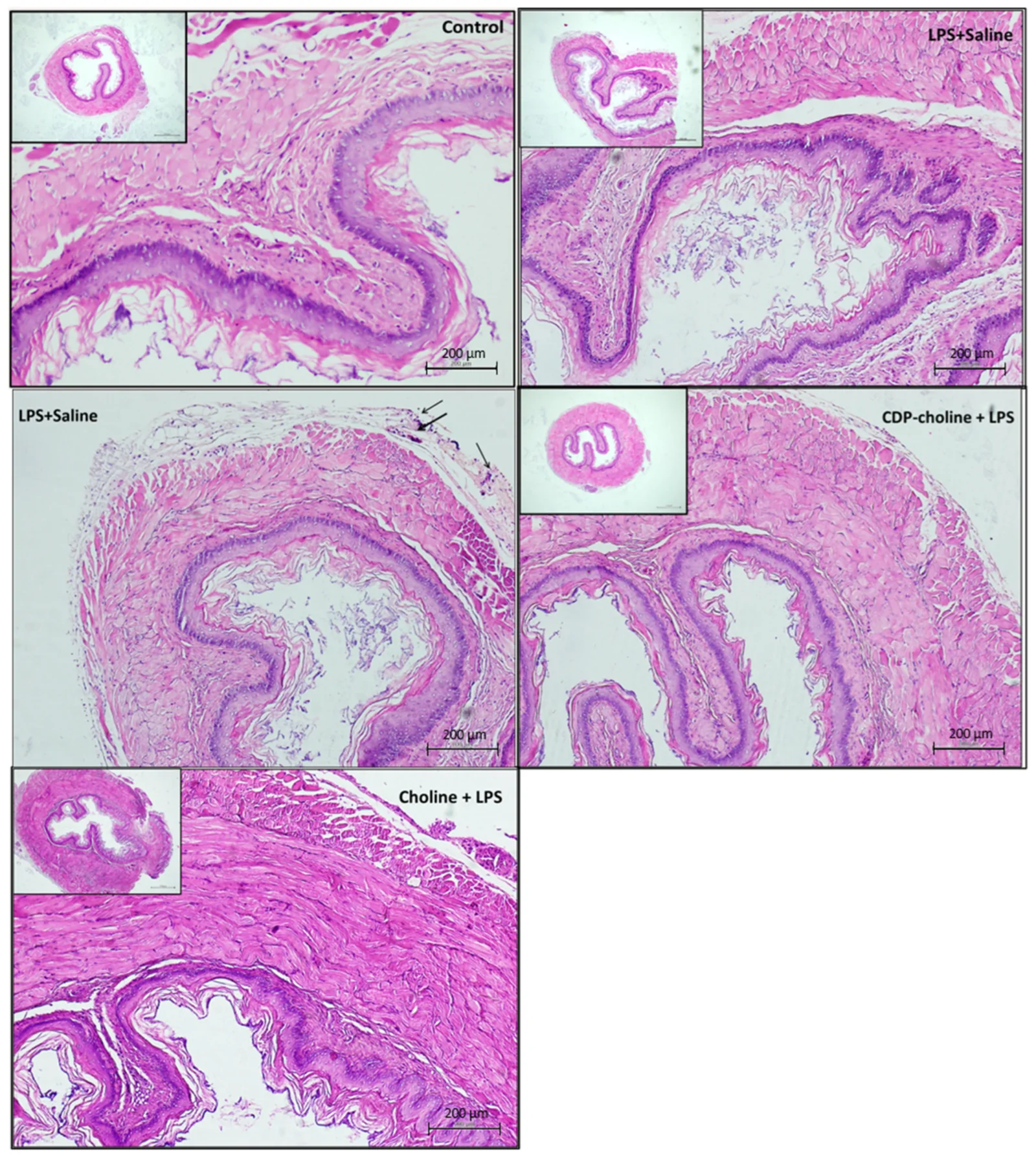
Effects of CDP-choline and choline on histopathological changes in the esophagus. Representative light-microscopic images of H&E staining in esophageal tissue of experimental groups in esophagus. (Scale bars = 200 µm). Black arrows in LPS + saline group indicate inflammatory cell infiltration in the tunica adventitia.

Histopathological examination revealed normal esophageal architecture in the control group. The LPS group showed generally preserved tissue architecture with increased inflammatory cell infiltration in the tunica adventitia compared with the control group. The LPS + CDP-choline group exhibited preserved esophageal architecture without inflammation, whereas inflammatory foci were observed in several animals in the LPS + choline group (Figure 1, Table 2).

**Figure 2:**
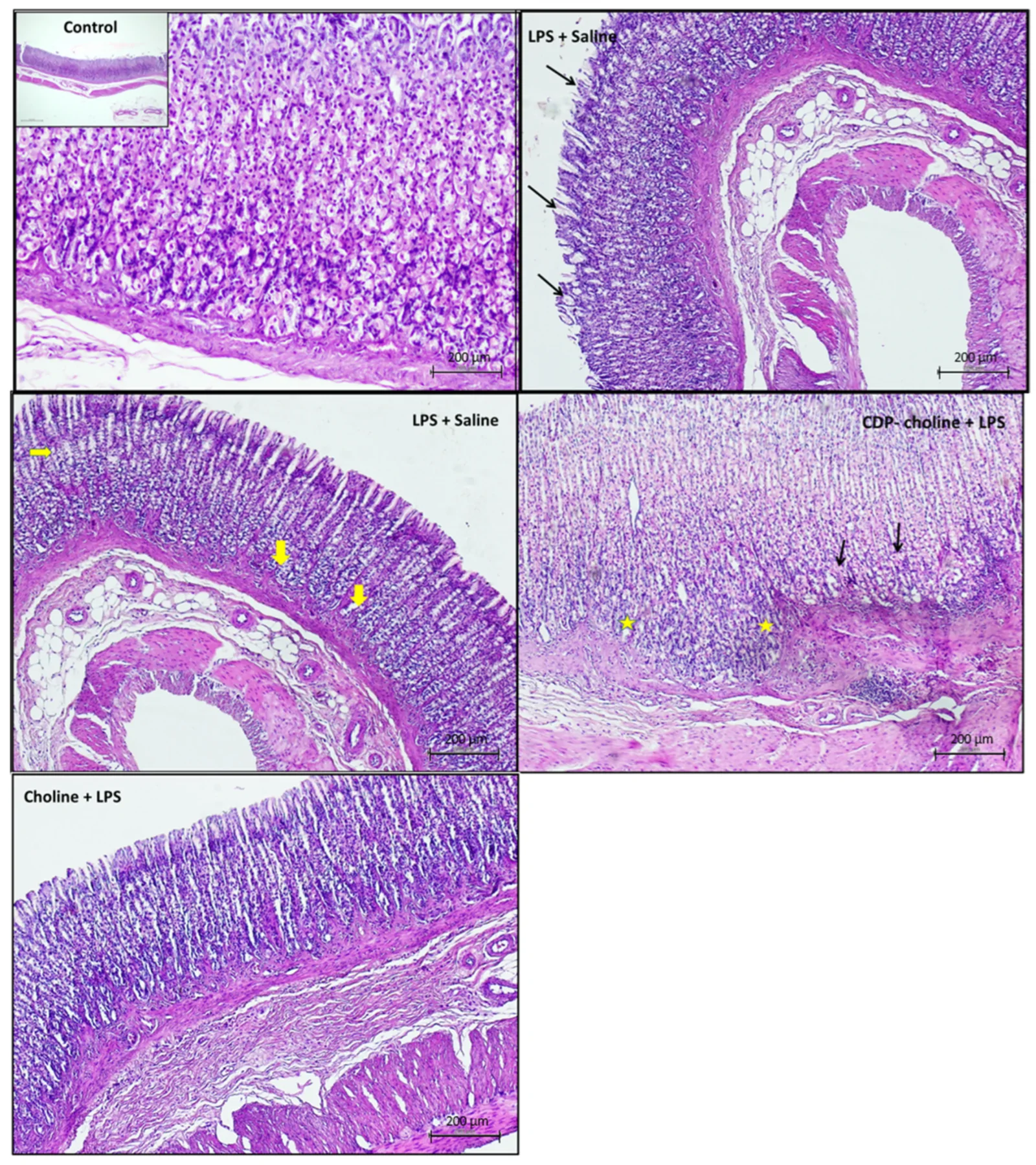
Effects of CDP-choline and choline on histopathological changes in the stomach. Representative light-microscopic images of H&E staining in stomach tissue of experimental groups in stomach. (Scale bars = 200 µm). Black arrows in the LPS + saline group indicate erosion of the surface mucous epithelium. Yellow arrows in the LPS + saline group indicate vacuolization of gastric mucous neck cells, parietal cells, and zymogenic (chief) cells. Black arrows in the CDP-choline + LPS group indicate preserved eosinophilic parietal cells. Yellow stars in the CDP-choline + LPS group indicate preserved zymogenic (chief) cells.

Assessment of stomach tissues indicated that the control group exhibited normal gastric histology. In the LPS group, marked vacuolization was identified in the neck and deep glandular epithelial cells of the gastric glands. Furthermore, erosion of the surface mucous epithelial cells was apparent. Diffuse vacuolization was additionally present in the mucous neck cells as well as in the parietal and zymogen cells. In the LPS + CDP-choline group, the gastric tissue retained normal histological appearance, and eosinophilic parietal cells and zymogen cells were clearly preserved with normal morphology. Likewise, the LPS + choline group demonstrated gastric histological findings comparable to those of the saline control group, without remarkable pathological alterations (Figure 2).

**Figure 3:**
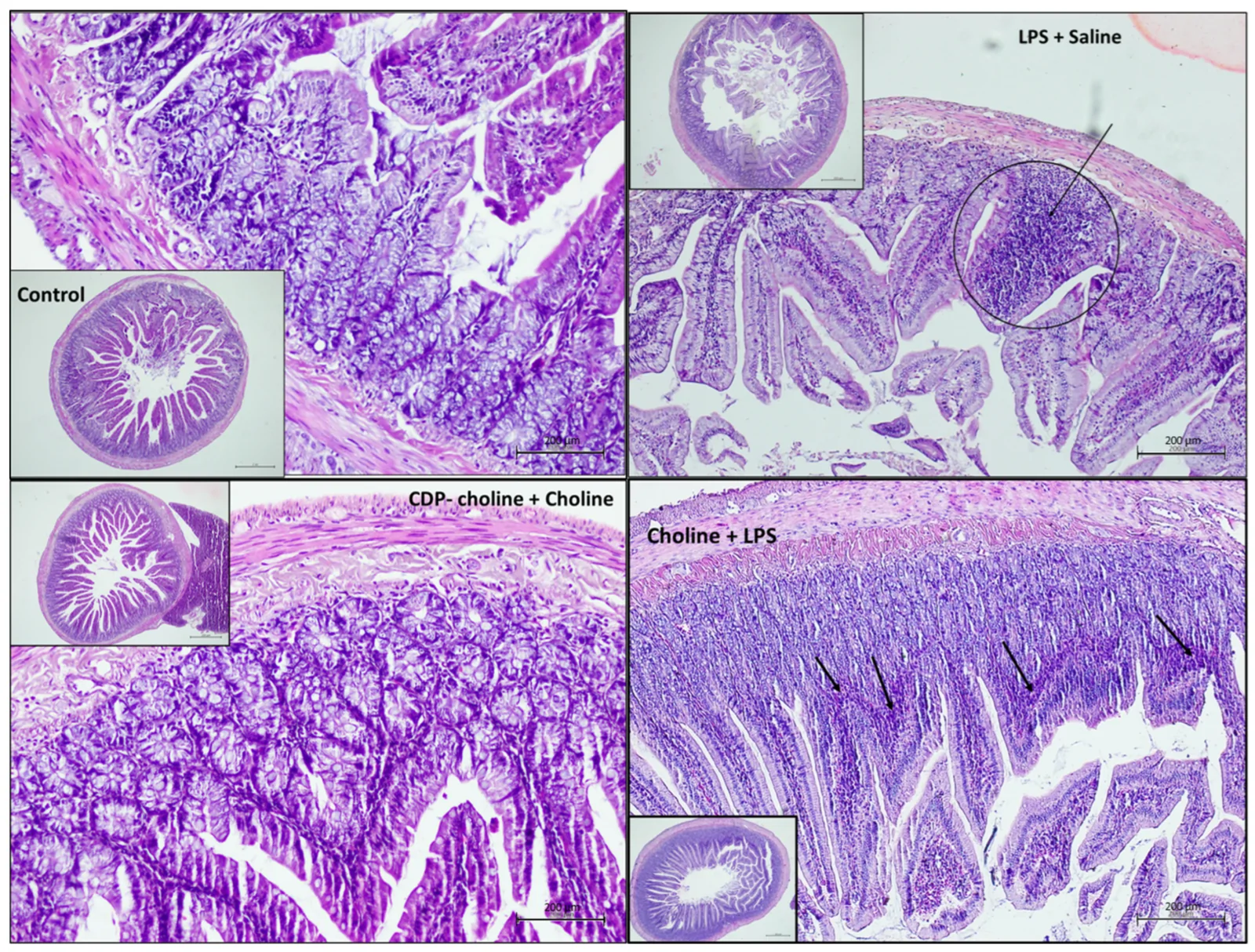
Effects of CDP-choline and choline on histopathological changes in the duodenum. Representative light-microscopic images of H&E staining in duodenum tissue of experimental groups in duodenum. (Scale bars = 200 µm). Black arrow and circled area in the LPS + saline group indicate prominent inflammatory cell infiltration in the lamina propria. Black arrows in the choline + LPS group indicate focal areas of inflammatory cell infiltration within the connective tissue of the lamina propria.

Duodenal tissues demonstrated normal small intestinal histological architecture in all tissue layers of the control group. In contrast, the LPS group exhibited areas characterized by prominent inflammatory cell infiltration within the intestinal tissue. The LPS + CDP-choline group maintained normal histological organization throughout all intestinal layers without obvious pathological findings. In the LPS + choline group, focal inflammatory regions were identified within the connective tissue of the lamina propria (Figure 3).

**Figure 4:**
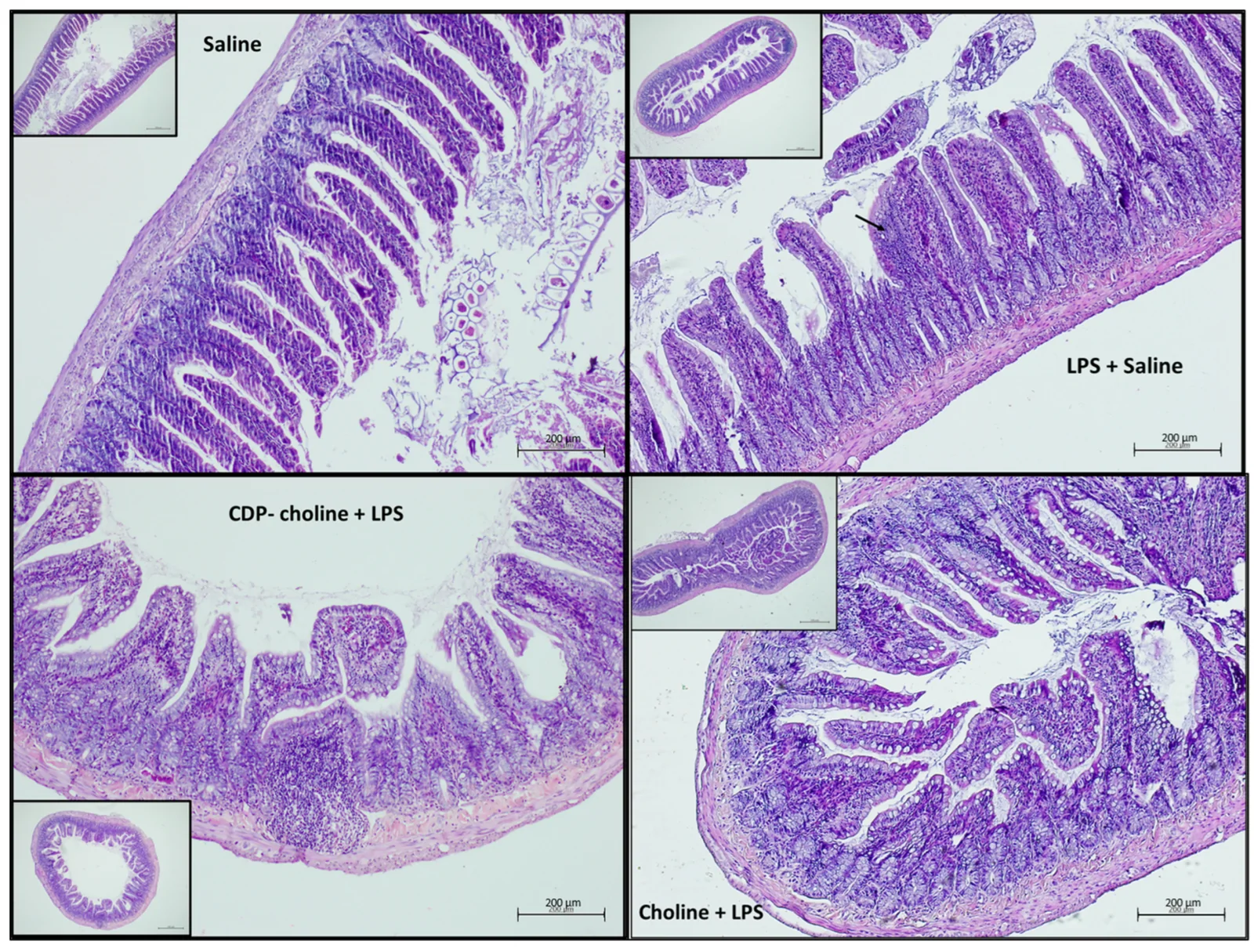
Effects of CDP-choline and choline on histopathological changes in the cecum. Representative light-microscopic images of H&E staining in cecum tissue of experimental groups in cecum. (Scale bars = 200 µm). Black arrows in the LPS + saline group indicate inflammatory cell infiltration in the lamina propria beneath the epithelium.

Cecal tissues showed that the control group preserved normal histological structure, whereas in the LPS group, inflammatory regions were detected in the lamina propria beneath the epithelial layer. LPS + CDP-choline and LPS + choline groups displayed normal histological morphology without significant pathological findings (Figure 4).

**Figure 5:**
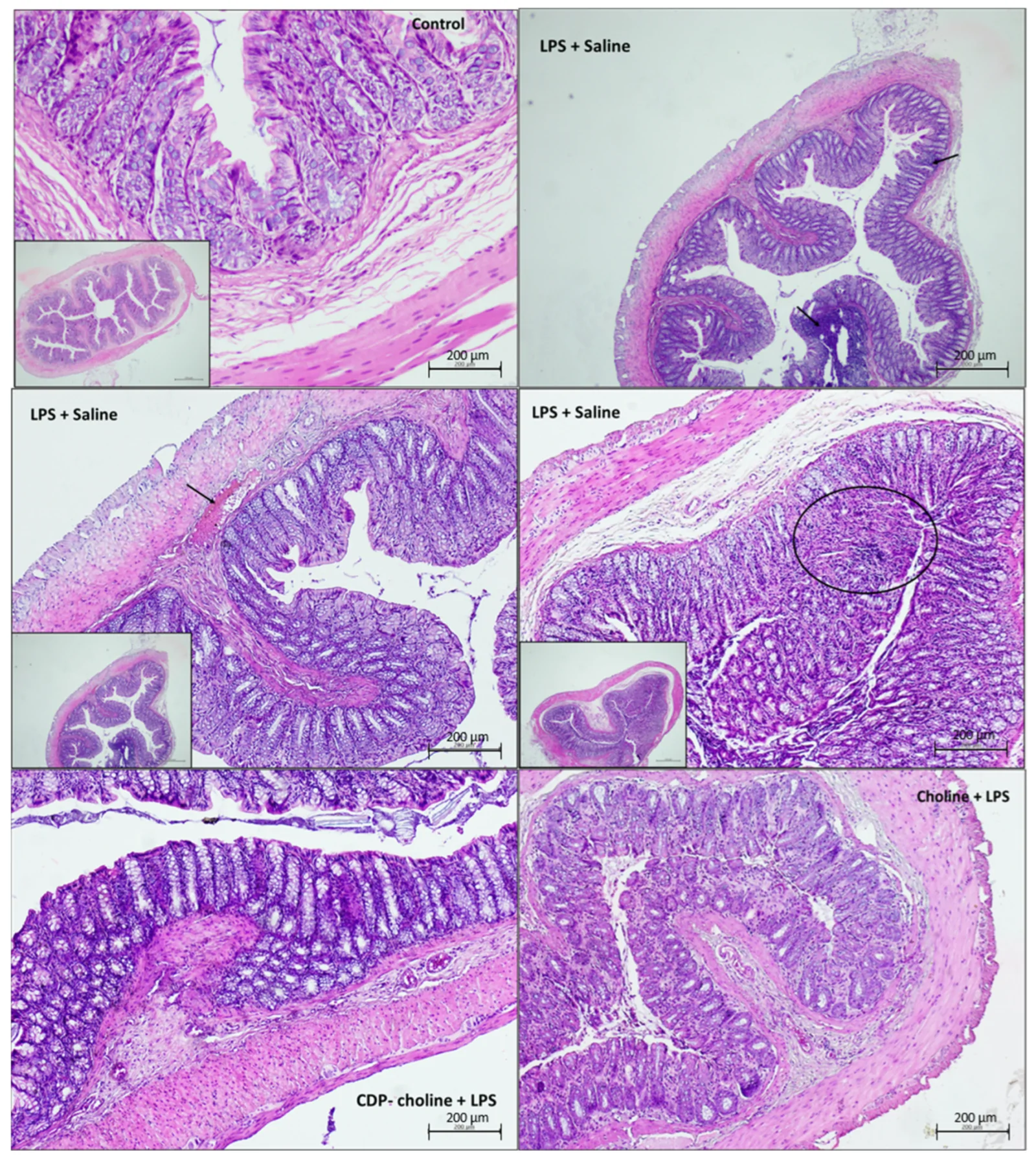
Effects of CDP-choline and choline on histopathological changes in the colon. Representative light-microscopic images of H&E staining in colon tissue of experimental groups in colon. (Scale bars = 200 µm). Black arrow in the LPS + saline group indicates inflammatory cell infiltration in the lamina propria of the mucosa. Black arrow in the LPS + saline group indicates a focal area of hemorrhage in the submucosa. Black arrow and circled area in the LPS + saline group indicate erosion of the surface epithelium.

Colon tissues demonstrated that the control group exhibited normal colonic architecture. In the LPS group, inflammatory regions were present in the lamina propria of the mucosal layer. Additionally, focal hemorrhagic areas were observed within the submucosa, and erosive lesions were apparent in the superficial epithelial layer. The histological findings of the LPS + CDP-choline group were consistent with those of the saline control group, with preservation of normal tissue architecture throughout all layers. Conversely, the LPS + choline group showed histopathological features resembling those of the LPS + saline group, including marked erosion of the surface epithelial cells within the tunica mucosa (Figure 5).

### 3.3. Effects of CDP-Choline and Choline on TNF-α Levels

**Figure 6:**
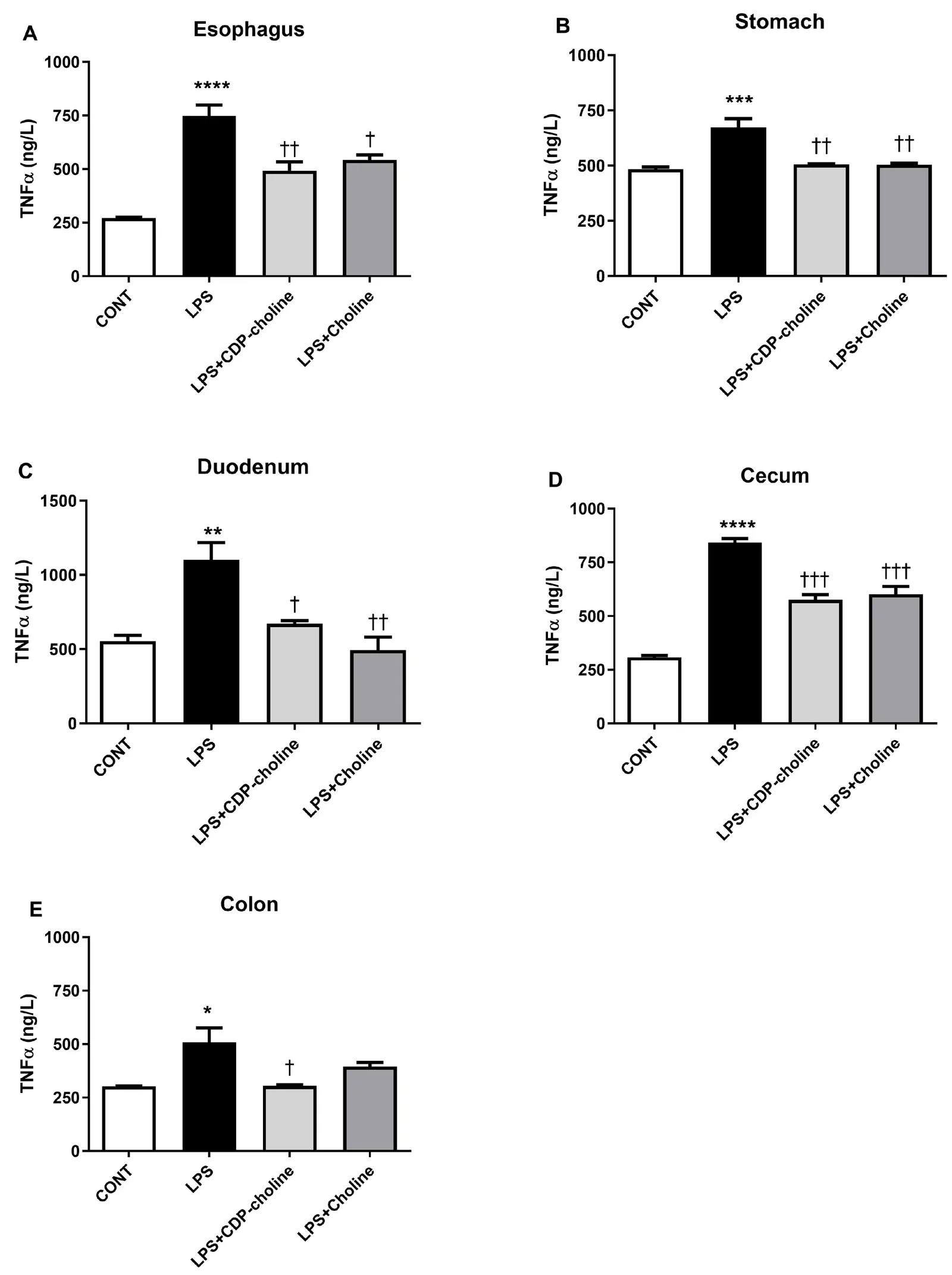
Effects of CDP-choline and choline on TNF-α levels in GI tissues following LPS-induced endotoxemia in rats. TNF-α concentrations were measured in the esophagus, stomach, duodenum, cecum, and colon. Data are presented as mean ± SEM. * *p* < 0.05, ** *p* < 0.01, *** *p* < 0.001, **** *p* < 0.0001 vs. Control; † *p* < 0.05, †† *p* < 0.01, ††† *p* < 0.001 vs. LPS. LPS, lipopolysaccharide.

TNF-α levels in all examined GI regions were increased in LPS group compared to controls, with the highest elevations observed in the duodenum, cecum, and esophagus (*p* < 0.001) (Supplementary Table S1). TNF-α levels in the cecum, colon, and esophagus were significantly decreased in LPS + CDP-choline group compared with LPS (*p* < 0.001 or *p* < 0.01). In LPS + Choline group, mild and region-dependent reductions were detected. In the cecum, TNF-α levels decreased with both treatment groups compared to LPS group (Figure 6).

### 3.4. Effects of CDP-Choline and Choline on IL-6 Levels

**Figure 7:**
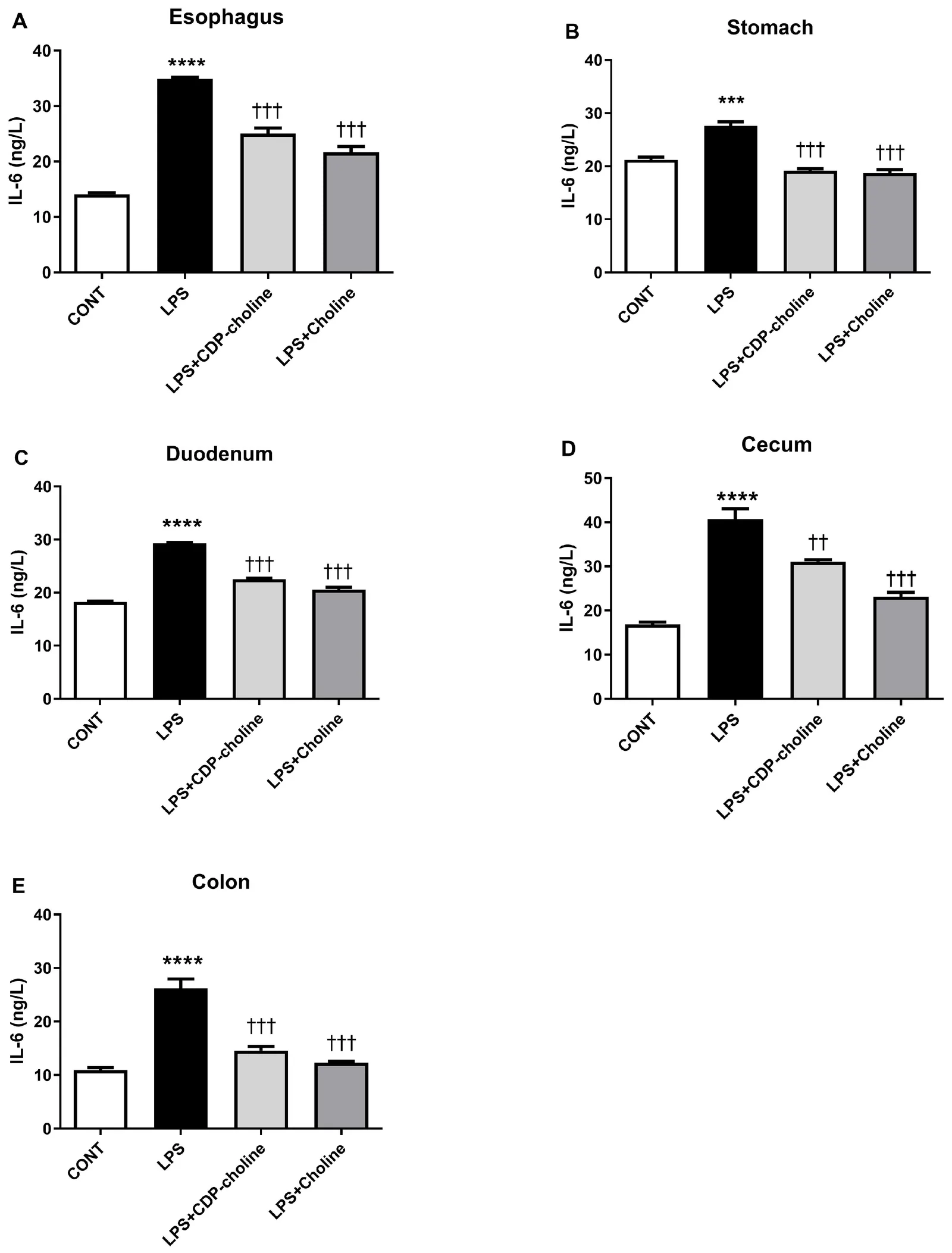
Effects of CDP-choline and choline on IL-6 levels in GI tissues following LPS-induced endotoxemia in rats. IL-6 concentrations were measured in the esophagus, stomach, duodenum, cecum, and colon. Data are presented as mean ± SEM. **** *p* < 0.0001, *** *p* < 0.001 vs. Control; †† *p* < 0.01, ††† *p* < 0.001 vs. LPS. LPS, lipopolysaccharide.

IL-6 levels in all examined GI tissues significantly increased in LPS group compared with controls, with the most pronounced elevations observed in the cecum, duodenum, and esophagus (*p* < 0.001). Both CDP-choline and choline treatment significantly reduced IL-6 levels in all five gastrointestinal regions examined—the esophagus, stomach, duodenum, cecum, and colon—compared with the LPS group (*p* < 0.001 or *p* < 0.01) (Figure 7).

### 3.5. Effects of CDP-Choline and Choline on 5-HT Levels

**Figure 8:**
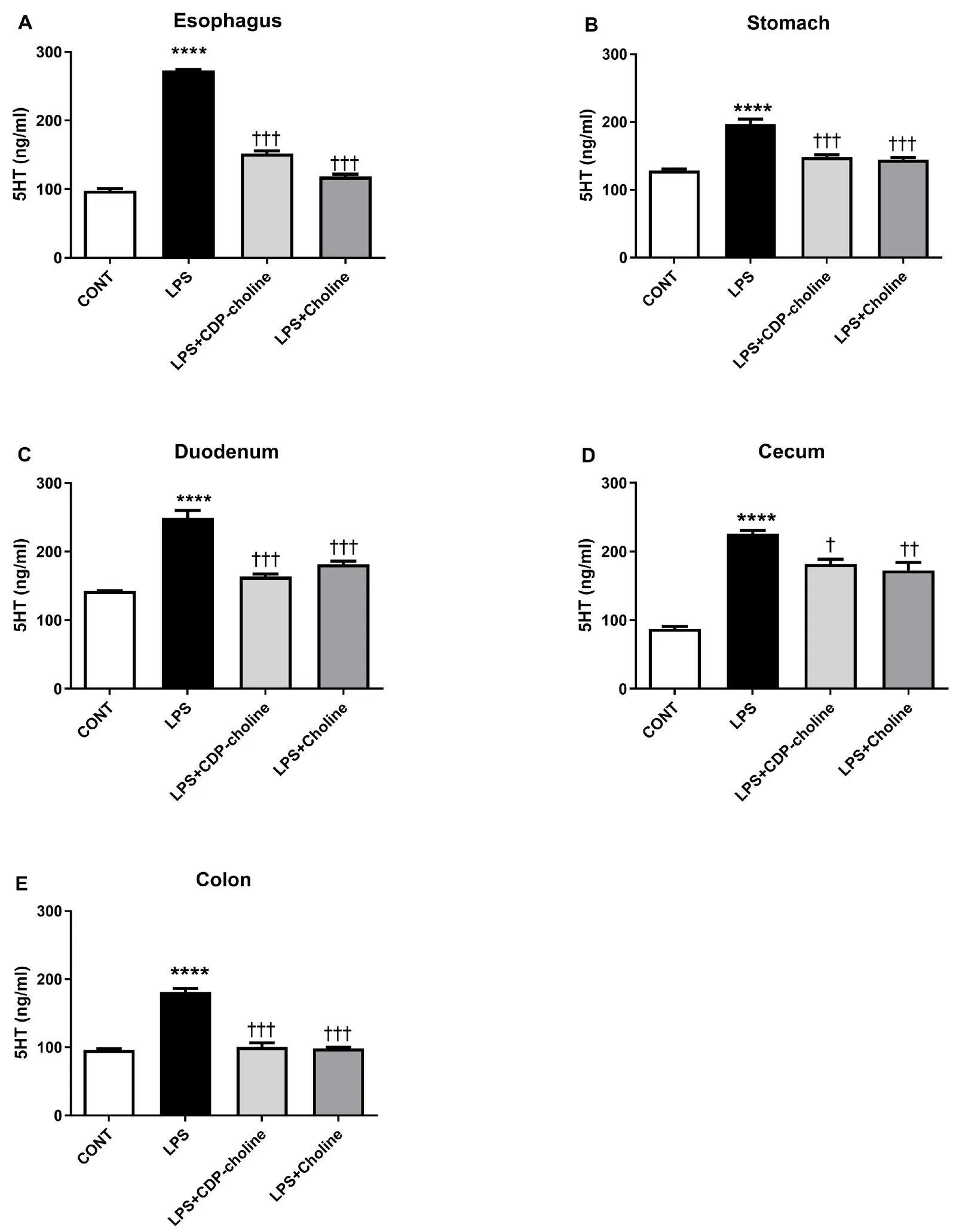
Effects of CDP-choline and choline on 5-HT levels in GI tissues during LPS-induced endotoxemia in rats. 5-HT concentrations were measured in the esophagus, stomach, duodenum, cecum, and colon. Data are presented as mean ± SEM. **** *p* < 0.0001 vs. Control; † *p* < 0.05, †† *p* < 0.01, ††† *p* < 0.001 vs. LPS. LPS, lipopolysaccharide.

LPS administration significantly increased 5-HT levels across all examined GI regions compared with controls. Both CDP-choline and choline treatment significantly reduced LPS-induced 5-HT elevations in a region-dependent manner. In the esophagus and colon, both treatments markedly attenuated 5-HT levels toward control values. The cecum also showed pronounced LPS-induced increases, with significant reductions observed following both CDP-choline and choline administration (Figure 8).

### 3.6. Effects of CDP-Choline and Choline on NE Levels

**Figure 9:**
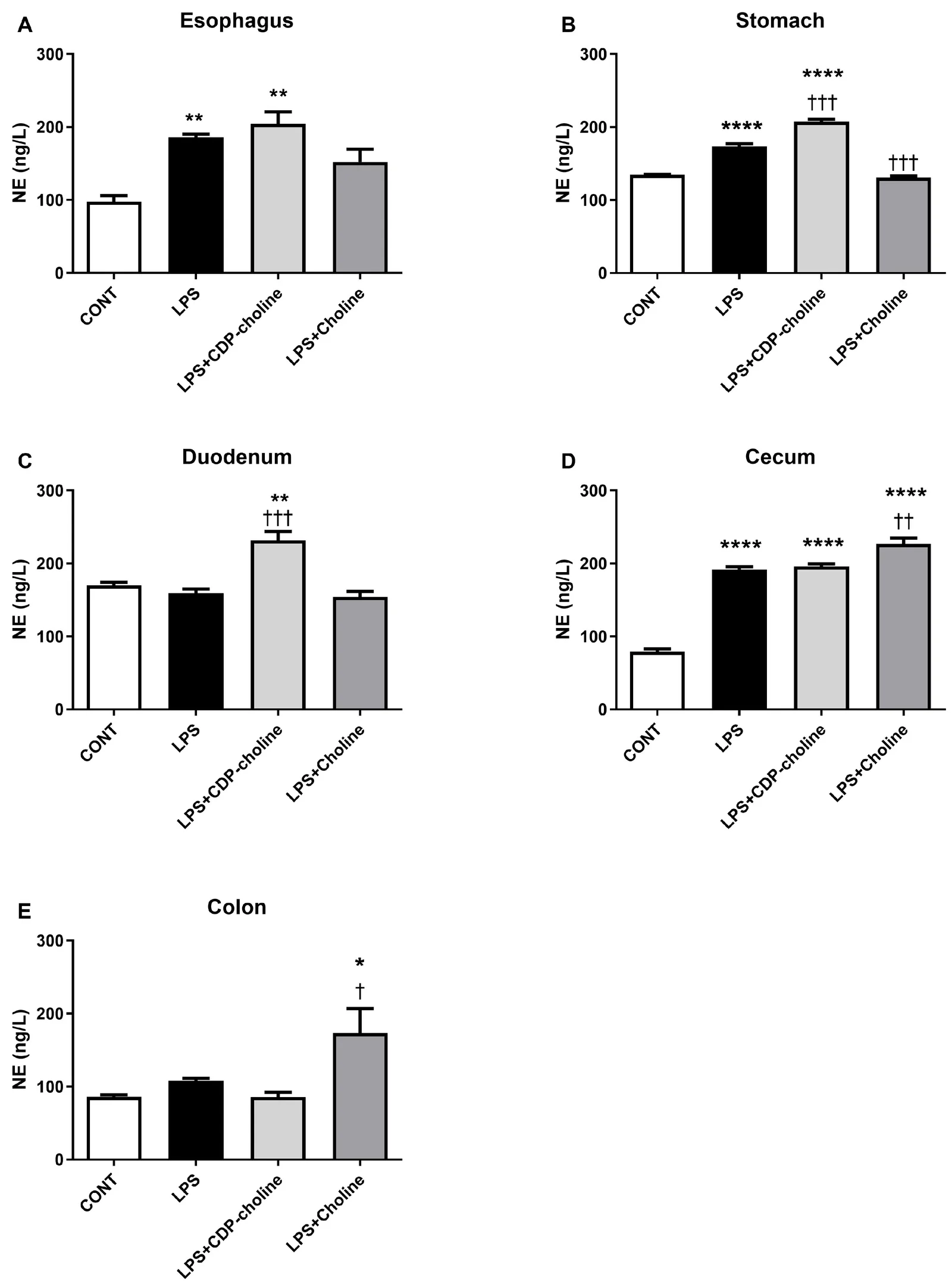
Effects of CDP-choline and choline on NE levels in GI tissues during LPS-induced endotoxemia in rats. NE concentrations were measured in the esophagus, stomach, duodenum, cecum, and colon. Data are presented as mean ± SEM. * *p* < 0.05, ** *p* < 0.01, **** *p* < 0.0001 vs. Control; † *p* < 0.05, †† *p* < 0.01, ††† *p* < 0.001 vs. LPS. LPS, lipopolysaccharide.

In the esophagus, LPS increased NE levels compared with controls, with modest and non-significant reductions observed following CDP-choline or choline co-treatment. In the stomach, LPS significantly elevated NE levels, which were attenuated by choline treatment, whereas CDP-choline further increased NE levels. In the duodenum, LPS alone did not significantly affect NE; however, CDP-choline administration resulted in a significant NE elevation, while choline-treated groups remained comparable to controls. The cecum showed NE increases following LPS and CDP-choline treatments, whereas choline significantly reduced NE levels compared with LPS alone. In the colon, NE levels did not differ significantly among groups (Figure 9).

### 3.7. Effects of CDP-Choline and Choline on DA Levels

**Figure 10:**
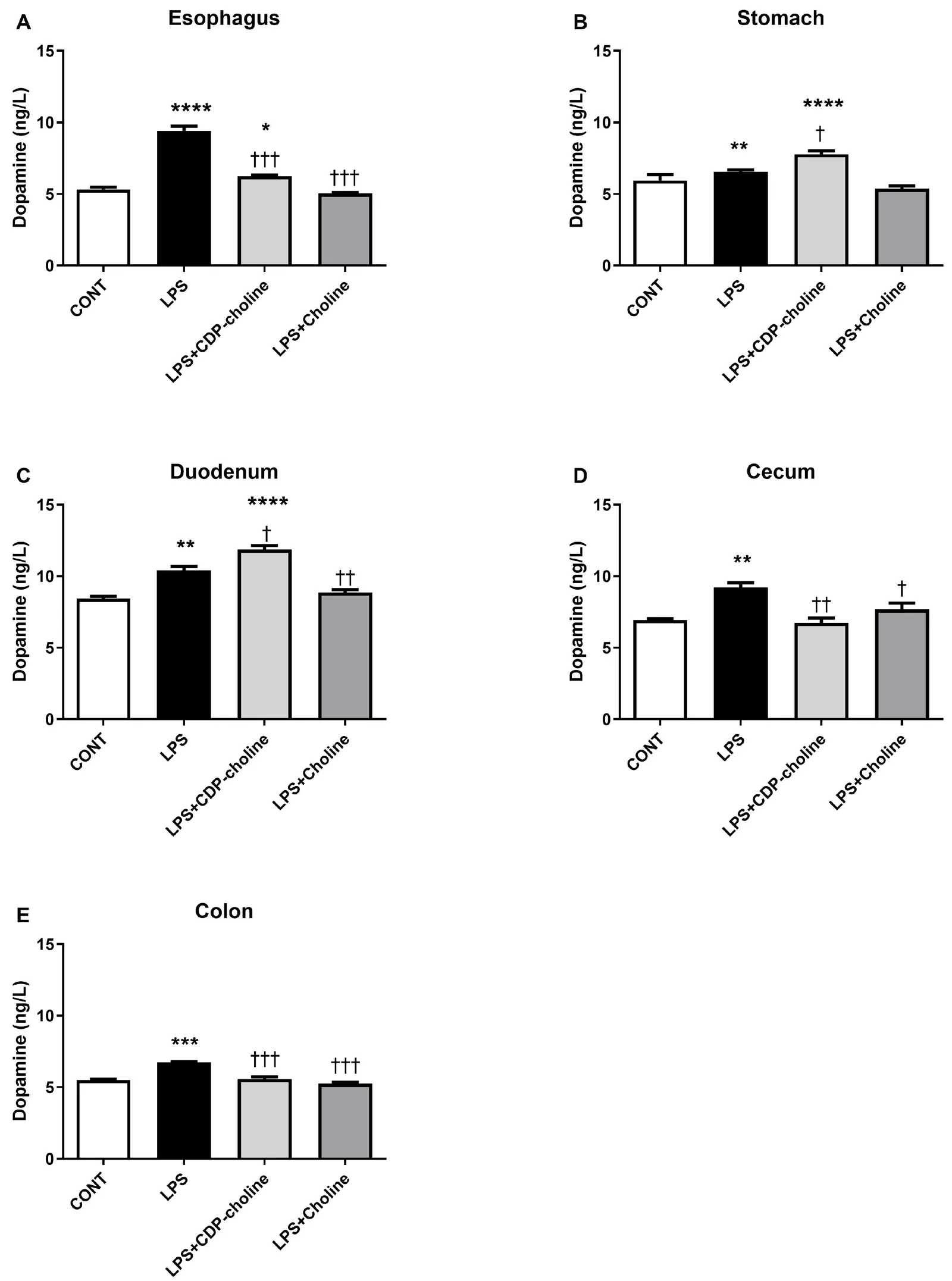
Effects of CDP-choline and choline on dopamine (DA) levels in GI tissues during LPS-induced endotoxemia in rats. DA concentrations were measured in the esophagus, stomach, duodenum, cecum, and colon. Data are presented as mean ± SEM. * *p* < 0.05, ** *p* < 0.01, *** *p* < 0.001, **** *p* < 0.0001 vs. Control; † *p* < 0.05, †† *p* < 0.01, ††† *p* < 0.001 vs. LPS. LPS, lipopolysaccharide.

In the esophagus, duodenum, cecum, and colon, LPS significantly increased DA levels compared with controls. Both CDP-choline and choline treatments attenuated LPS-induced DA elevations in the esophagus, cecum, and colon, with values approaching those of controls. In the duodenum, CDP-choline further increased DA levels relative to LPS alone, whereas choline treatment significantly reduced DA levels compared with the LPS group. In contrast, gastric DA levels showed no significant differences among experimental groups (Figure 10).

### 3.8. Effects of CDP-Choline and Choline on ChAT Levels

**Figure 11:**
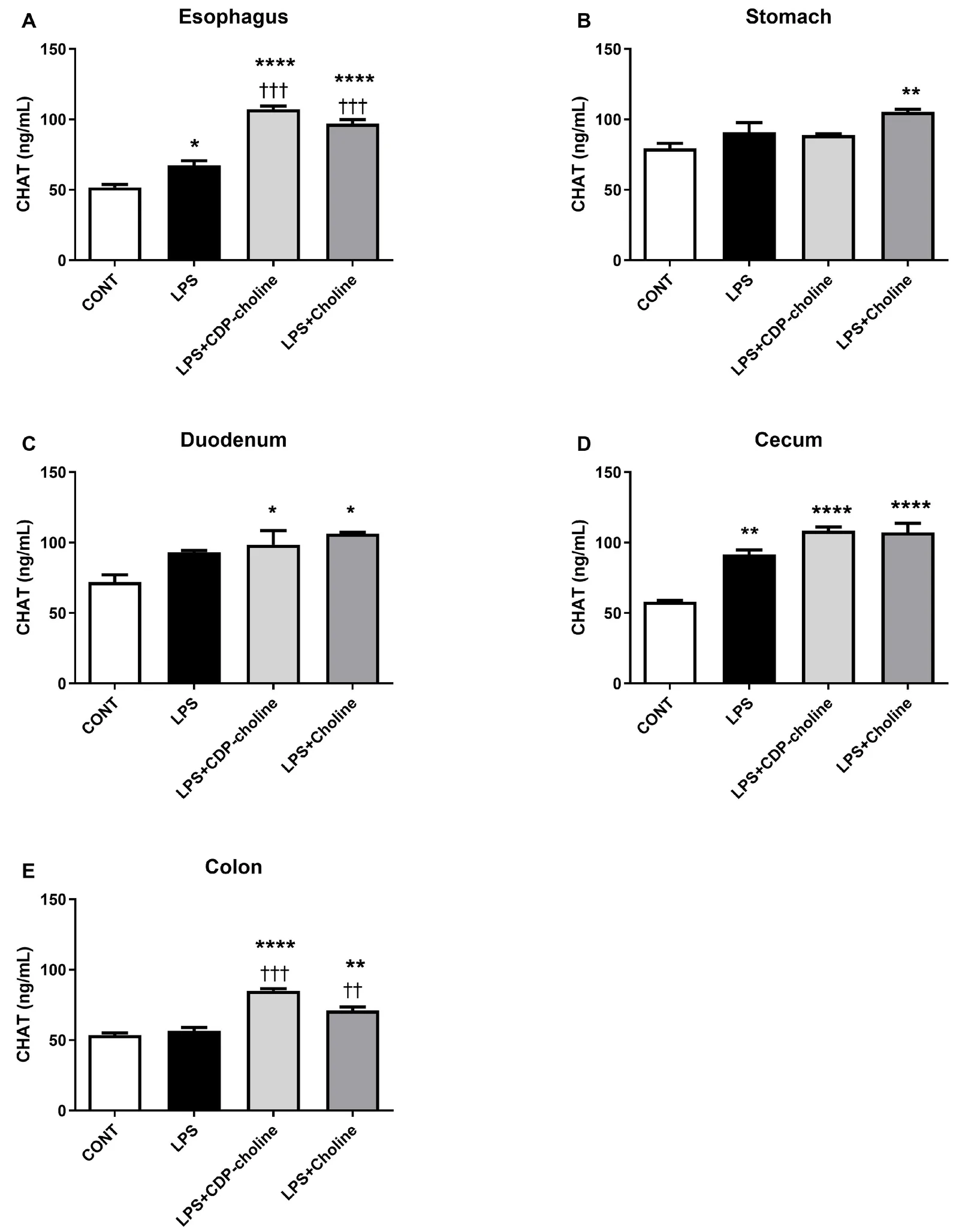
Effects of CDP-choline and choline on choline acetyltransferase (ChAT) levels in GI tissues during LPS-induced endotoxemia in rats. ChAT concentrations were measured in the esophagus, stomach, duodenum, cecum, and colon. Data are presented as mean ± SEM. * *p* < 0.05, ** *p* < 0.01, **** *p* < 0.0001 vs. Control; †† *p* < 0.01, ††† *p* < 0.001 vs. LPS. LPS, lipopolysaccharide.

LPS administration did not significantly alter ChAT levels in most GI tissues except esophagus and cecum compared with controls. In contrast, CDP-choline treatment markedly increased ChAT levels, particularly in the esophagus, cecum, and colon (*p* < 0.001 vs. LPS), with moderate increases observed in the duodenum and stomach. Choline treatment also elevated ChAT levels in selected tissues, notably the colon and esophagus, but these effects were consistently less pronounced than those induced by CDP-choline. In the colon, ChAT levels differed significantly between CDP-choline and choline-treated groups (Figure 11).

## 4. Discussion

Sepsis is a severe inflammatory condition characterized by marked systemic physiological and metabolic disturbances. In the present study, LPS-induced endotoxemia was associated with increased clinical severity scores and body weight loss. The mechanisms underlying the weight change were not directly assessed. Choline and CDP-choline, as cholinergic system-acting agents and acetylcholine precursor molecules, have emerged as potential therapeutic candidates for endotoxemia due to their demonstrated organ-protective effects, vascular function preservation, and anti-inflammatory properties [23,24].

LPS administration at a sublethal dose (10 mg/kg) successfully induced marked endotoxemia, as evidenced by significantly elevated sepsis scores and pronounced body weight reduction, validating the model for investigating gastrointestinal inflammatory responses [6,17]. Sepsis scores in the LPS group remained consistently elevated at both 6 and 24 h, demonstrating sustained endotoxemia severity across the observation period. At 6 h, neither CDP-choline nor choline significantly reduced LPS-induced sepsis scores. At 24 h, CDP-choline significantly attenuated sepsis scores compared with the LPS group, whereas choline produced a greater reduction in mean scores but with substantially higher inter-animal variability, largely attributable to one animal that maintained elevated scores throughout the observation period. These findings indicate that LPS administration induced the intended acute systemic inflammatory phenotype under the experimental conditions used. This provided a basis for examining region-specific gastrointestinal inflammatory responses and associated changes in tissue ChAT and monoamine levels [19,25,26].

LPS administration induced distinct histopathological changes across GI regions. In the esophagus, LPS caused increased inflammatory cell infiltration in the tunica adventitia, while gastric tissues showed marked vacuolization and surface erosions. The histopathological findings describe tissue-specific manifestations of LPS-induced gastrointestinal injury. Because different pathological features were assessed in different organs, the numerical scores do not provide a common measure of injury severity across gastrointestinal regions. Treatment-associated differences were therefore interpreted within each tissue relative to its corresponding control and LPS groups. These findings do not establish a quantitative ranking of organ susceptibility or demonstrate that treatment effects differ significantly between organs [27,28]. Endotoxemia disrupts mucosal integrity through oxidative stress, inflammatory cytokine release, and epithelial injury [29]. Histopathological observations were interpreted within each gastrointestinal tissue under the dosing regimens examined. Qualitative differences in representative sections should not be interpreted as evidence of superior histopathological efficacy of either compound. The mechanisms underlying the observed tissue changes were not directly established in this study. CDP-choline treatment effectively preserved normal tissue architecture across all regions examined, particularly in the stomach, duodenum, and colon, whereas choline demonstrated substantial protection in esophageal and gastric tissues but only partial preservation in intestinal segments. These protective effects likely reflect CDP-choline-mediated suppression of local pro-inflammatory cytokine production and stabilization of cellular membranes through enhanced phosphatidylcholine availability, mechanisms consistent with the biochemical and cholinergic findings presented before [6,21,30].

Pro-inflammatory cytokine responses showed clear regional variation. Following LPS administration, TNF-α levels increased significantly across all gastrointestinal regions (*p* < 0.001), with the most pronounced elevations observed in the duodenum, cecum, and esophagus. CDP-choline significantly reduced TNF-α levels in the cecum, colon, and esophagus (*p* < 0.001 or *p* < 0.01), with moderate reductions in the stomach and duodenum, whereas choline produced milder and less consistent reductions across regions. These cytokine elevations are consistent with activation of Toll-like receptor 4 (TLR4) and MyD88/NF-κB signaling pathways [31], with possible additional involvement of the Wnt/β-catenin pathway [32]. Both CDP-choline and choline significantly reduced IL-6 levels in the duodenum, cecum, colon, and esophagus (*p* < 0.001 or *p* < 0.01); pro-inflammatory cytokine responses varied across gastrointestinal regions. CDP-choline significantly reduced TNF-α levels in the esophagus, cecum, and colon relative to LPS, whereas the responses to choline varied by region. Both treatments significantly reduced IL-6 levels in all five gastrointestinal regions examined. These findings describe the effects of CDP-choline sodium salt dihydrate at 375 mg/kg and choline chloride at 90 mg/kg under the present experimental conditions. Differences in the magnitude or statistical significance of responses relative to LPS do not, by themselves, demonstrate a significant difference between the treatments. Although the regimens supplied approximately comparable theoretical amounts of choline, systemic and tissue exposure were not measured. The observed response patterns, therefore, cannot be attributed to superior bioavailability or interpreted as evidence of intrinsic pharmacological superiority. The pronounced sensitivity of intestinal segments, particularly the duodenum, cecum, and colon, may be related to their greater immune cell density, more extensive lymphoid tissue, and higher baseline inflammatory activity compared with upper gastrointestinal segments. This regional vulnerability is supported by the histopathological findings, as the strongest cytokine elevations in the duodenum and cecum corresponded with greater inflammatory infiltration in these tissues [33].

Tissue ChAT content showed region-dependent responses to treatment. CDP-choline increased ChAT levels, particularly in the esophagus, cecum, and colon, while choline also increased ChAT levels in selected regions. These findings demonstrate changes in the abundance of an enzyme involved in acetylcholine synthesis. Previous experimental work has established a role for ChAT-expressing, acetylcholine-producing cells in neural regulation of inflammatory responses [5]. However, ChAT enzymatic activity and acetylcholine concentrations were not measured in the present study; therefore, increased ChAT content cannot establish enhanced acetylcholine production or functional cholinergic signaling. Although α7nAChR-dependent suppression of inflammatory cytokines has been demonstrated experimentally [34], the accompanying cytokine reductions in our study do not establish that ChAT changes mediated the anti-inflammatory effects. Specifically, α7nAChR activation and downstream signaling were not assessed, and pathway blockade was not performed. Involvement of the cholinergic anti-inflammatory pathway, therefore, remains a hypothesis requiring direct investigation. These region-specific ChAT responses suggest that CDP-choline enhances local acetylcholine synthesis, a pattern consistent with, but not directly demonstrating, engagement of α7nAChR-mediated anti-inflammatory signaling via the cholinergic anti-inflammatory pathway (CAP) [34,35], as acetylcholine release and receptor activation were not assessed in this study.

Endotoxemia disrupts intestinal immune homeostasis, promoting sustained release of TNF-α and IL-6 and persistent macrophage activation, which collectively cause gastrointestinal inflammatory injury and altered neurotransmitter regulation [36,37,38]. Inflammatory signaling impairs monoamine synthesis, metabolism, and tissue availability, exacerbating neurochemical imbalance during systemic inflammation [37,39,40]. These interconnected inflammatory-neurochemical mechanisms help explain the marked monoaminergic dysregulation observed across gastrointestinal segments, which is discussed in detail below, and provide mechanistic rationale for examining the capacity of cholinergic agents to modulate these responses.

LPS-induced endotoxemia caused pronounced disturbances in gastrointestinal monoamine content, reflecting altered neurochemical balance during systemic inflammation. LPS significantly increased tissue concentrations of 5-HT, DA, and NE across multiple regions, with the most marked changes in the duodenum and cecum. These intestinal segments have high immune cell density and active neurochemical regulation, making them particularly sensitive to inflammatory stimuli [4,41]. Because most gastrointestinal serotonin is synthesized locally, inflammatory disruption of mucosal integrity likely contributes to region-specific monoaminergic dysregulation [42,43,44]. The comprehensive elevation of serotonin, dopamine, and norepinephrine across multiple gastrointestinal regions suggests a coordinated disruption of enteric monoaminergic systems, likely reflecting inflammation-induced changes in monoamine synthesis, metabolism, and transporter function.

5-HT levels increased significantly across all regions following LPS, representing a consistent neurochemical response to endotoxemia. Both CDP-choline and choline significantly attenuated LPS-induced 5-HT elevations in a region-dependent manner, with the most pronounced reductions in the esophagus and colon, and marked suppression in the cecum, where baseline LPS-induced elevations were most severe. These findings align with previous reports showing that cholinergic pathway activation modifies serotonin levels during inflammatory stress [10], potentially through modulation of enterochromaffin cell function and serotonin transporter activity [11,43].

DA levels increased significantly in the esophagus, duodenum, cecum, and colon following LPS, with minimal changes in the stomach. Both treatments attenuated DA elevations in the esophagus, cecum, and colon, approaching control levels, demonstrating successful normalization of dopaminergic dysregulation in these regions. However, in the duodenum, CDP-choline paradoxically further increased DA levels while choline significantly reduced them, suggesting region-specific differences in cholinergic modulation of dopaminergic function. LPS alters tissue DA levels depending on the tissue and timing of exposure [12,13]. These observations are consistent with reports showing that choline supplementation modulates dopaminergic availability in inflammatory conditions [14]. The increase in duodenal DA following CDP-choline treatment may reflect restoration or compensatory activation of local enteric dopaminergic signaling rather than a pro-inflammatory response, particularly because cytokine levels and histopathological findings improved simultaneously. Nevertheless, the divergent duodenal responses to CDP-choline and choline may also involve differential engagement of cholinergic receptor subtypes with opposing effects on dopaminergic regulation, although this hypothesis requires further investigation using selective receptor antagonists.

NE levels showed the most variable and region-specific responses among the three monoamines examined. LPS increased NE in the esophagus, stomach, duodenum, and cecum, with minimal effects in the colon. Treatment responses differed markedly by region: in the esophagus and colon, both CDP-choline and choline produced minimal NE modulation; in the stomach, choline reduced NE while CDP-choline paradoxically increased it; in the duodenum, CDP-choline induced significant NE elevation while choline maintained control levels; in the cecum, both LPS and CDP-choline elevated NE while choline reduced it. These regional response patterns highlight the complexity of sympathetic-enteric nervous system interactions during endotoxemia and suggest that norepinephrine may reflect both inflammatory and adaptive processes depending on tissue location [45]. The differential NE modulation by CDP-choline versus choline, dissociated from their comparable suppression of pro-inflammatory cytokines, raises the possibility that NE elevation represents a distinct adaptive response to cholinergic stimulation rather than a pathological process [46]. CDP-choline’s effects on NE likely reflect its broader role in phospholipid synthesis, membrane stabilization, and cholinergic modulation, supporting its capacity to counteract inflammation-associated neurochemical disturbances [35,47,48]. In contrast, choline produced weaker and more variable effects, indicating limited capacity to normalize noradrenergic alterations during acute endotoxemia.

The concurrent changes in tissue ChAT, cytokines, and monoamines raise the possibility of interactions between inflammatory and neurochemical responses during endotoxemia. Experimental evidence of communication between intestinal neurons and macrophages provides a biological basis for considering such interactions [4]. However, the co-occurrence of these changes in our study does not establish a causal relationship or demonstrate coordinated regulation through a common cholinergic mechanism. Altered monoamine handling is another possible explanation; for example, LPS has been shown to modify serotonin transporter function in human intestinal epithelial cells [37]. Cholinergic receptor signaling and monoamine synthesis, release, uptake, and metabolism were not directly examined in the present study, and tissue monoamine concentrations alone cannot distinguish among these processes. Accordingly, our findings demonstrate treatment-associated changes in gastrointestinal inflammatory and neurochemical profiles rather than restoration of functional neurochemical homeostasis.

Cholinergic signaling has been proposed in the literature to modulate monoaminergic homeostasis through several mechanisms, including (1) direct modulation of monoamine-producing neurons via acetylcholine receptor subtypes, (2) indirect effects of cytokine production on enteric neuron monoamine metabolism, and (3) potential modulation of monoamine transporter function [29,49,50]. None of these specific mechanisms was directly investigated in the present study. The co-occurrence of cholinergic marker alterations and monoamine changes observed here is consistent with, but does not establish, a functional relationship between these systems. Collectively, these findings support the hypothesis that enhanced cholinergic capacity through CDP-choline administration may contribute to gastrointestinal neurochemical homeostasis, potentially via coordinated modulation of inflammatory cytokine production and monoaminergic regulation—a hypothesis that remains to be directly tested in future mechanistic studies (e.g., receptor antagonism, transporter assays, or direct acetylcholine measurement).

### Limitations

This study has several limitations that should be acknowledged. First, this study was conducted exclusively in female Sprague–Dawley rats. Although the inclusion of females contributes information on this population, the absence of a male comparison group prevents assessment of sex-dependent differences in susceptibility to endotoxemia or responsiveness to CDP-choline and choline. Experimental evidence indicates that biological sex and gonadal hormone signaling can influence inflammatory responses to LPS, with the magnitude of these differences depending on the dose and outcome assessed [51]. These factors could therefore influence the inflammatory environment in which the gastrointestinal histopathological and neurochemical responses were measured. However, their contribution to the present findings cannot be determined, and neither the magnitude nor the direction of the treatment effects should be assumed to be identical in males. Furthermore, the estrous-cycle stage was not determined, preventing assessment of whether cycle-related hormonal variation contributed to differences within or between groups. This represents an unmeasured potential source of variability rather than an established explanation for the observed results. Future studies should include both sexes and record estrous-cycle stage to evaluate these influences directly.

Second, the modest sample size of six animals per group limits the precision of treatment-effect estimates and may reduce the ability to detect smaller differences, particularly for variable neurochemical outcomes. Individual observations may also have a substantial influence on group means and variability estimates. Consequently, non-significant comparisons should not be interpreted as evidence of equivalence or absence of a biological effect. Measurements from multiple gastrointestinal tissues and technical assay replicates do not increase the number of independent animals. Together with the numerous outcomes examined, these considerations support interpreting the findings as exploratory.

Third, biochemical analyses were limited to tissue-level measurements of inflammatory cytokines and monoamines; the following mechanistic components were not assessed: (1) α7 nicotinic acetylcholine receptor (α7nAChR) expression and phosphorylation; (2) monoamine transporter (SERT, DAT, and NET) expression; (3) Toll-like receptor 4 (TLR4) signaling and expression; (4) neurotransmitter release rates; and (5) functional gastrointestinal motor and secretory outcomes. Therefore, the observed biochemical changes should be interpreted as alterations in tissue inflammatory and neurochemical content that are associated with, but do not directly prove, functional changes in neurotransmission, neuroimmune signaling, or gastrointestinal function. Estrous-cycle stage was not determined, preventing assessment of its potential contribution to variability in the measured outcomes. Future studies incorporating estrous-cycle staging would help clarify whether the responses to LPS, CDP-choline, and choline vary across cycle stages. The comparison between CDP-choline and choline is limited to the dosing regimens examined. Although the preparations supplied approximately comparable theoretical amounts of choline, systemic and tissue exposure were not measured, and only one dose of each compound was evaluated. Consequently, differences between treatments cannot establish intrinsically greater pharmacological efficacy. Furthermore, the contribution of the cytidine component of CDP-choline was not isolated experimentally and remains uncertain. This study assessed six biochemical markers across five GI regions alongside histopathological and clinical endpoints, without a prespecified primary outcome or correction for multiplicity across comparisons; correction was applied only within each test. Given the small sample size (*n* = 6/group), findings, particularly those near the significance threshold, should be interpreted as hypothesis-generating. Sepsis scores at 6 h and 24 h were analyzed as independent comparisons rather than with a formal repeated-measures approach, which future studies should consider. Confirmation requires larger studies with a prespecified primary outcome and an a priori sample-size calculation, including sufficient animals, to examine sex-by-treatment interactions.

Also, treatment was initiated before LPS administration; therefore, the observed effects reflect a pretreatment regimen that included a subsequent post-LPS dose. The study cannot distinguish the contributions of the two doses or establish efficacy when treatment is initiated only after endotoxemia has developed. Further studies using exclusively post-LPS administration are needed to evaluate therapeutic potential.

## 5. Conclusions

Acute LPS-induced endotoxemia induces marked, region-specific inflammatory and monoaminergic alterations within the gastrointestinal tract, particularly in the duodenum and cecum. Under the dosing regimens and experimental conditions examined, CDP-choline and choline modulated inflammatory cytokine levels, tissue ChAT content, and monoamine concentrations, with responses varying across gastrointestinal regions and endpoints. These findings do not establish intrinsic pharmacological superiority of either compound. Notably, norepinephrine responses were more heterogeneous than serotonin and dopamine responses, suggesting region-specific regulation of sympathetic and enteric noradrenergic signaling during endotoxemia. Acute LPS-induced endotoxemia was associated with region-specific gastrointestinal inflammatory, histopathological, and monoaminergic changes. Under the dosing regimens examined, CDP-choline and choline modified these outcomes and tissue ChAT content, with effects varying across regions and measured endpoints. These findings do not establish activation of the cholinergic anti-inflammatory pathway or a causal relationship between ChAT changes and cytokine reduction. Future investigations should focus on (1) determining whether CDP-choline effects extend to male and aged animals and chronic sepsis models; (2) directly assessing α7nAChR involvement using selective antagonists; (3) examining whether normalization of monoamine dysregulation translates to improved gastrointestinal motility and barrier function; (4) investigating the relative contributions of phospholipid synthesis and cholinergic pathway activation to CDP-choline’s anti-inflammatory effects; and (5) translating these experimental findings toward clinical evaluation in sepsis-associated gastrointestinal complications; (6) acetylcholine measurements; (7) ChAT activity assays; and (8) receptor-specific interventions.

## Figures and Tables

**Table 1:** Sepsis scores at 6 and 24 h after LPS administration.

	Sepsis Score at 6 h			Sepsis Score at 24 h		
Parameter	LPS	LPS + Choline	LPS + CDP-Choline	LPS	LPS + Choline	LPS + CDP-Choline
Appearance	2.08 ± 0.86	2.42 ± 1.02	2.50 ± 0.71	2.42 ± 1.16	0.67 ± 0.98	1.80 ± 0.45
Level of consciousness	2.25 ± 0.88	2.08 ± 0.97	2.50 ± 0.84	2.08 ± 0.75	0.58 ± 1.20	1.70 ± 0.45
Activity	2.58 ± 0.94	2.08 ± 1.52	2.50 ± 1.17	2.75 ± 1.08	0.50 ± 1.22	2.10 ± 0.84
Response to stimulus	2.42 ± 0.83	1.58 ± 1.09	2.17 ± 1.06	2.25 ± 0.74	0.58 ± 0.73	1.80 ± 0.65
Eyes	2.17 ± 0.41	1.75 ± 1.12	1.75 ± 0.94	2.33 ± 0.61	0.58 ± 0.58	1.30 ± 0.74
Respiratory rate	2.33 ± 0.82	1.58 ± 1.09	1.58 ± 0.82	2.00 ± 1.05	0.67 ± 0.61	1.10 ± 0.65
Respiratory quality	2.42 ± 0.58	1.42 ± 0.75	1.75 ± 0.42	2.42 ± 1.02	0.25 ± 0.42	1.10 ± 0.22
TOTAL	16.3± 1.1 **	12.9 ± 2.8 **	14.7 ± 2.0 **	15.7 ± 1.7 **	3.8 ± 2.2 ††	9.8 ± 0.9 †

Endotoxemia severity scores following LPS administration and treatment with CDP-choline or choline. Effects of LPS-induced sepsis and choline-based interventions on sepsis scores (6 h and 24 h). Data are expressed as mean ± SEM values. ** *p* < 0.01 vs. control, † *p* < 0.05 and †† *p* < 0.01 vs. LPS.

**Table 2:** Effects of CDP-choline and choline on histopathological injury scores.

Tissue	Group	Mean ± SD
Esophagus	Control	0.17 ± 0.41
	LPS	0.50 ± 0.55
	LPS + CDP-choline	0.00 ± 0.00
	LPS + choline	1.00 ± 0.89
Stomach	Control	0.17 ± 0.41
	LPS	1.17 ± 0.75
	LPS + CDP-choline	0.17 ± 0.41
	LPS + choline	0.17 ± 0.41
Duodenum	Control	0.17 ± 0.41
	LPS	2.00 ± 0.63 **
	LPS + CDP-choline	0.17 ± 0.41 ^††^
	LPS + choline	0.50 ± 0.55
Cecum	Control	0.00 ± 0.00
	LPS	1.50 ± 0.55 **
	LPS + CDP-choline	0.00 ± 0.00 ^††^
	LPS + choline	0.33 ± 0.52 ^†^
Colon	Control	0.33 ± 0.52
	LPS	2.33 ± 0.52 *
	LPS + CDP-choline	0.00 ± 0.00 ^††^
	LPS + choline	0.50 ± 0.84 ^†^

Inflammation was graded as 0 (no inflammation), 1 (scattered small inflammatory foci), 2 (scattered larger inflammatory foci accompanied by hemorrhage), or 3 (diffuse inflammation); one score was assigned per animal for each tissue (*n* = 6 per group). Data are presented as mean ± SD. Groups were compared within each tissue using the Kruskal–Wallis test followed by Dunn’s post hoc test. * *p* < 0.05, ** *p* < 0.01 vs. control; ^†^ *p* < 0.05, ^††^ *p* < 0.01 vs. LPS. LPS, lipopolysaccharide.

## Data Availability

The raw data supporting the conclusions of this article will be made available by the authors on request.

## Supplementary Materials

The following supporting information can be downloaded at: https://www.mdpi.com/article/10.3390/biomedicines14102271/s1, Table S1: Tissue biomarker concentrations by treatment group and gastrointestinal region.

## Abbreviations

5-HT (5-Hydroxytryptamine); Ach (Acetylcholine; CAP (Cholinergic Anti-Inflammatory Pathway); CDP-choline (Cytidine-5′-Diphosphocholine); ChAT (Choline Acetyltransferase); DA (Dopamine); IL-1β (Interleukin-1 Beta); IL-6 (Interleukin-6); TNF-α (Tumor Necrosis Factor Alpha); LPS (Lipopolysaccharide); NE (Norepinephrine); NF-κB (Nuclear Factor Kappa B); SERT (Serotonin Transporter); TLR4 (Toll-Like Receptor 4); α7nAChR (Alpha-7 Nicotinic Acetylcholine Receptor).
