# Supplementary material for: Effects of CDP-Choline and Choline on Gastrointestinal Inflammatory and Monoaminergic Responses in an LPS-Induced Rat Endotoxemia Model
Source: Biomedicines. 2026 Oct 8;14(10):2271. doi: 10.3390/biomedicines14102271 (PMC13702001; doi:10.3390/biomedicines14102271)
Supplement: Supplementary file 1 [file biomedicines-14-02271-s001.zip › biomedicines-4587481-supplementary.pdf]

Supplementary Table S1. Tissue biomarker concentrations by treatment group and gastrointestinal region.

Biomarker	GI Region	Control	LPS	LPS + CDP-choline	LPS + Choline
**TNF-α (pg/mg protein)**	Esophagus	271.11 ± 9.36	748.99 ± 87.79	493.22 ± 70.53	543.02 ± 39.87
	Stomach	483.83 ± 16.57	673.12 ± 68.72	505.27 ± 5.73	504.49 ± 11.46
	Duodenum	554.32 ± 68.09	1101.82 ± 201.50	672.12 ± 34.73	494.90 ± 148.81
	Cecum	307.67 ± 15.40	842.23 ± 31.87	575.81 ± 40.53	600.95 ± 63.82
	Colon	302.51 ± 2.99	508.30 ± 118.00	304.93 ± 8.33	395.15 ± 33.87
**IL-6 (pg/mg protein)**	Esophagus	14.08 ± 0.51	34.89 ± 0.54	25.05 ± 1.71	21.64 ± 1.84
	Stomach	21.20 ± 0.91	27.63 ± 1.31	19.16 ± 0.67	18.71 ± 1.11
	Duodenum	18.25 ± 0.29	29.24 ± 0.36	22.54 ± 0.36	20.56 ± 0.77
	Cecum	16.85 ± 0.97	40.71 ± 4.09	31.07 ± 0.78	23.16 ± 1.72
	Colon	10.95 ± 0.74	26.22 ± 2.99	14.56 ± 1.44	12.32 ± 0.46
**5-HT (ng/mg protein)**	Esophagus	97.98 ± 5.21	272.92 ± 2.47	151.99 ± 6.88	118.45 ± 6.03
	Stomach	128.36 ± 4.05	197.03 ± 12.83	147.92 ± 6.29	144.33 ± 5.66
	Duodenum	142.55 ± 1.05	249.20 ± 18.81	163.70 ± 6.66	181.44 ± 8.05
	Cecum	87.57 ± 5.93	225.91 ± 8.40	181.65 ± 12.49	172.68 ± 20.16
	Colon	96.07 ± 2.98	181.07 ± 9.29	100.27 ± 10.74	98.44 ± 3.01
**ChAT (ng/mg protein)**	Esophagus	51.81 ± 3.51	67.31 ± 5.76	107.16 ± 4.04	97.04 ± 5.04
	Stomach	79.47 ± 6.33	91.10 ± 11.65	88.79 ± 1.96	105.36 ± 3.27
	Duodenum	72.03 ± 8.83	93.22 ± 2.13	98.56 ± 17.42	106.32 ± 1.62
	Cecum	58.12 ± 1.52	91.56 ± 5.74	108.46 ± 4.59	107.29 ± 11.20
	Colon	53.64 ± 2.59	56.59 ± 4.35	85.01 ± 2.71	71.02 ± 4.39
**NE (ng/mg protein)**	Esophagus	97.53 ± 15.03	185.97 ± 7.22	204.45 ± 28.48	152.02 ± 30.68
	Stomach	134.61 ± 1.11	173.26 ± 6.66	207.49 ± 5.47	130.80 ± 4.19
	Duodenum	169.82 ± 7.89	159.36 ± 9.40	231.84 ± 20.74	154.28 ± 13.23
	Cecum	79.13 ± 6.45	191.66 ± 6.74	195.85 ± 6.45	226.71 ± 14.34
	Colon	85.79 ± 5.38	107.85 ± 6.15	85.55 ± 11.42	173.27 ± 58.24
**DA (ng/mg protein)**	Esophagus	5.32 ± 0.27	9.41 ± 0.58	6.25 ± 0.14	5.03 ± 0.11
	Stomach	5.95 ± 0.71	6.54 ± 0.24	7.79 ± 0.40	5.37 ± 0.35
	Duodenum	8.43 ± 0.28	10.40 ± 0.49	11.86 ± 0.49	8.86 ± 0.35
	Cecum	6.94 ± 0.16	9.22 ± 0.56	6.73 ± 0.60	7.67 ± 0.77
	Colon	5.51 ± 0.11	6.74 ± 0.07	5.57 ± 0.27	5.25 ± 0.19

Values are expressed as mean ± SD. Abbreviations: TNF-α, tumor necrosis factor-alpha; IL-6, interleukin-6; 5-HT, serotonin; ChAT, choline acetyltransferase; NE, norepinephrine; DA, dopamine; LPS, lipopolysaccharide; SD, standard deviation.
